# Methodological quality of studies of end-stage renal disease risks in lupus nephritis

**DOI:** 10.1186/ar4663

**Published:** 2014-09-18

**Authors:** Maria Tektonidou, Abhijit Dasgupta, Michael Ward

**Affiliations:** 1 School of Medicine, University of Athens, Greece; 2 National Institute of Arthritis and Musculoskeletal and Skin Diseases, National Institutes of Health, Bethesda, MD, USA

## Background

Variations in methodological quality can affect the results of individual studies and of systematic reviews. We examined the adequacy of patient descriptions, representativeness, and follow-up information in studies included in a systematic review of risks of end-stage renal disease (ESRD) in patients with lupus nephritis.

## Methods

We search Medline, Embase, and the Cochrane Database from their inceptions to 31 December 2013 for studies that reported on ESRD in adults with lupus nephritis. We included all observational studies and long-term clinical trials with a minimum of 12 months of follow-up and 10 patients that reported specific data on the development of ESRD. Two authors independently assessed study quality using a modification of the Newcastle Ottawa scale, and rated studies on 10 items in three areas: adequacy of description of the cohort (items 1 to 3); representativeness (items 4 to 7); and adequacy of follow-up information (items 8 to 10).

## Results

The literature search yielded 1,852 articles, of which 174 articles met our inclusion criteria. These included 132 observational studies and 42 clinical trials. The proportion of studies meeting each quality measure, stratified by study design, is presented in Table 1. Among observational studies, the median number of measures satisfied per study was 5 (range 2 to 9), and among clinical trials was 4 (range 2 to 7). There was no correlation between publication year and number of measures satisfied for observational studies (*r *= 0.14), but recent trials tended to satisfy more quality measures (*r *= 0.32; *P *= 0.04).

**Table 1:** 

Quality measure	Observational studies (*n *= 132)	Clinical trials (*n *= 42)
1. ACR criteria for SLE used	88.3	88.1
2. Measure of renal function included	77.2	95.2
3. Treatments described	84.1	100
4. Community-based study	11.3	Not applicable
5. Inception cohort	40.1	11.9
6. Renal biopsy not required for inclusion	37.1	35.7
7. Patients with chronic kidney disease not excluded	80.3	23.8
8. Losses to follow-up described	24.2	42.8
9. Losses to follow-up < 20%	18.9	30.9
10. Data reported using Kaplan-Meier curves	36.3	9.5

## Conclusions

While both observational studies and clinical trials generally provided good clinical descriptions of the cohorts, few provided adequate data on follow-up. The representativeness of observational studies was low. The improvement in trial quality over time may be due to the development of standardized protocols and the institution of reporting standards, which might also serve to enhance the reporting of observational studies.

